# Investigation of the Consumer Perspective on Leisure in Mental Health Inpatient Units

**DOI:** 10.1177/15394492231175067

**Published:** 2023-06-02

**Authors:** Jessica Levick, Kieran Broome, Marion Gray, Theo Theodoros, Thomas Morrison, Manaan Kar Ray

**Affiliations:** 1 School of Health and Medical Sciences, University of Southern Queensland, Ipswich, Australia; 2 Good to Better, Imbil, Queensland, Australia; 3 Metro South Addiction and Mental Health Services, Queensland Health, Woolloongabba, Australia

**Keywords:** leisure, public health, mental health

## Abstract

This study aimed to explore the barriers to engagement in activity and consumer satisfaction in inpatient settings. Participants were current inpatient consumers and completed an online anonymous survey. This included the Mental Health Satisfaction Improvement Program (MHSIP), Leisure Boredom Scale (LBS), and the Checklist of Leisure Interests and Participation (CLIP). A total of 57 participants partially completed the survey with 41 completed responses. Participants reported several barriers to engagement, including lack of staff, limited social engagement, limited range of activity, and a lack of resources. Most participants reported to be either “very satisfied” (24.24%) or “somewhat satisfied” (36.36%) with the level of activity offered. Participants reported to be bored due to a limited occupational range offered in the mental health inpatient unit. Participants identified the need for assistance in the facilitation of activity.

## Introduction

Leisure can be an activity that is salutogenic (health-creating) and beneficial for one’s well-being (Caldwell, 2005). Leisure activity can assist in generating purpose and assist people with recovery from mental health issues (Craik & Pieris, 2006). In this study, we will explore the perspective of consumers regarding the availability and satisfaction of leisure activity in mental health inpatient units (MHIUs).

MHIUs often have limited occupational opportunities to enhance consumers’ recovery (Antonysamy, 2013; Marshall et al., 2020). Occupational opportunities refer to the opportunities to participate in meaningful occupation which can be due to the constraints of a person’s physical or social environment which can lead to occupational deprivation (Whiteford et al., 2020).

At times, time pressures and self-perceived priority of other activities or tasks in other occupational areas (such as productivity) can create an imbalance in leisure or free time (Yazdani et al., 2018). For people with mental health issues, Craik and Pieris (2006) highlighted that having adequate time was critical for leisure engagement. Some participants reported leisure activities as a regular part of their routine, while others used them reactively to avoid stress (Craik and Pieris, 2006). Encouraging consumers to reflect on their leisure profile and explore meaningful alternatives can be used as a therapeutic modality and an opportunity to open discussion on health-promoting practices (Hammell, 2004; Leufstadius, 2017; Leufstadius et al., 2009). Often, hospitalization can affect someone’s ability to engage in leisure activities from consumers’ typical occupational profile, forcing them to engage in foreign or personally uninteresting activities that they typically would not do in the community (Foye et al., 2020).

Dahlen et al. (2004) established a link between boredom and the external or physical environment. Poorly designed environments can perpetuate the experience of boredom and maladaptive aggressive and sensation-seeking behaviors. This aligns with broader research linking limited occupational opportunity or range of activity and consumer reported boredom (Folke et al., 2018; Foye et al., 2020; Marshall et al., 2020). Consumer boredom can be related to a perception of a monotonous environment, lack of goals or drive, and inability to gain a sense of excitement or enjoyment (Bowser et al., 2018). Interestingly, their research suggests that boredom in institutional settings (specifically forensic settings) can be from a lack of skills to participate in leisure rather than a limited range of opportunities to engage (Bowser et al., 2018). The finding indicated barriers to engagement were intrinsic motivation, exacerbated mental health issues, aggression, boredom, and lack of sleep; a restrictive environment, lack of daily responsibilities; and a lack of meaningful activity on offer (Bowser et al., 2018). A variety of studies have highlighted the lack of physical activity (Korge & Nunan, 2018) or a variety of meaningful activities are offered in MHIUs (Farnworth & Muñoz, 2009). Activities offered typically have a strong emphasis on arts and crafts (Ng et al., 2020). However, outdoor activities are rarely viable in inpatient units due to the built environment (Ng et al., 2020). There is limited research to explore the specific activities that consumers would like to participate in while on MHIUs and the overall impact this would have on consumer experience (Ng et al., 2020).

As a health care system, a cultural shift in the physical and social environment of MHIUs needs to occur to create occupational opportunity that is essential for mental health recovery (Whiteford et al., 2020). The literature suggests there are many barriers to engagement in meaningful occupations in acute settings. Little is known from the literature around what influence unit staff’s decisions to implement leisure activities. Some considerations to the challenges of implementing leisure activity may include perceived lack of time (Bowser et al., 2018; Marshall et al., 2020) (which could also be interpreted as perceived lack of priority), poor culture of activity engagement, devolution of responsibility for leisure facilitation among staff, and lack of access to resources (Levick, Broome, Ingram, et al., 2023). In practice, decisions around leisure offerings appear to be largely ad hoc (Cutler et al., 2021; Levick, Broome, Ingram, et al., 2023).

Some of the barriers found in the literature were a lack of allied health provided beyond business hours, a monotonous environment, and a limited range of activity provided. It is hypothesized that consumers will report low satisfaction and a high level of boredom when asked about their experience admitted to the MHIU.

This research aimed to answer the following questions:

1. Are consumers satisfied with the occupational opportunities available to them while admitted to an Australia MHIU?
2. What are some of the barriers and facilitators to consumers engaging in leisure activity?
3. Are there meaningful ways to improve the consumer experience while admitted to a MHIU and enable occupational opportunity?

## Materials and Methods

This study used a mixed-methods approach to explore consumers’ perspectives (Creswell et al., 2008) of leisure on MHIUs. Current consumers in MHIUs completed an online survey. Ethical approval was received from the Queensland Health, Metro South Health Ethics Committee (project number HREC/2021/QMS/76198), and the University of Southern Queensland Human Research Ethics Committee (project number H21REA304).

### Sample

Participants were recruited from the MHIUs at the Princess Alexandra Hospital, Woolloongabba, Queensland, Australia. Participants were surveyed across the four MHIUs, including a mixed gender unit, one female only unit, one male only unit, and a high dependency unit. Data were collected in mixed and single-gender (male or female) MHIUs. Consumers were invited to participate in the survey and participation was voluntary. Inclusion criteria required participants to be over 18 years old, with experience of being a consumer on a MHIU and having stayed overnight for more than 48 hr in a locked MHIU within the past 5 years. All participants who were recruited were admitted consumers to a locked MHIU. Participants were excluded from participating under the age of 18 (considered child, youth, or adolescent). This hospital has one occupational therapist that services all the MHIUs surveyed.

A sample size calculation was completed using the methods described by Charan and Biswas (2013). The standard normal variate selected was 1.96 (i.e., corresponding to a type 1 error of 5%). The sample size based on these parameters was 36 participants. As the survey is lengthy and is targeting acutely unwell consumers, this was considered adequate.

### Survey Design

Participants were asked to complete a survey through an online survey platform, Survey Monkey. The research participant information was provided at the beginning of the survey. Consumers were asked a question related to consent to continue. Participants’ IP addresses and names were not recorded for anonymity. Demographic data included information such as their age, geographical location (country, state/province, post/zip code), and mental health diagnosis. Consumers’ responses were anonymous, which allowed them to provide feedback on the inpatient unit without bias or judgment. We believe this assisted to provide authentic feedback.

Participants were provided with a definition of leisure to provide context and meaning to the questions. The definition provided was

leisure is considered an enjoyable activity that is not work or productive activity which you choose to participate in your spare time. Furthermore, leisure may also be activity that can be relaxing, fun and support health in a therapeutic way.

The surveys included a combination of tools and open-ended questions. This included The Mental Health Statistics Improvement Program (MHSIP) 21-Item Consumer Survey (Howard et al., 2003), Checklist of Leisure Interests and Participation (CLIP) (Levick, Broome, Oprescu, et al., 2023), Leisure Boredom Scale (LBS; Iso-Ahola & Weissinger, 1990), and open-ended questions.

The open-ended questions to gain consumers’ perspectives included the following:

- How did you keep yourself engaged in leisure on the inpatient unit or in the mental health wait room?
- What activities were available to you while you were inpatient or in the mental health wait room?
- What stopped you from engaging in leisure activities on the inpatient unit or in the mental health wait room?
- What changes would most improve your access to leisure activity on the MHIU or in the mental health wait room?

A multiple-choice question included the following:

- What is your understanding of your mental health diagnosis?
- How would you rate your ability to engage in leisure activity on the inpatient unit?
- Overall, how satisfied or dissatisfied were you with the level of leisure activities offered on the MHIU?

### Tools and Checklist Used

Two standardized tools and a checklist were used in this survey. The first tool was the MHSIP, which explored the contextual factors of participation. The MHSIP 21-Item Consumer Survey has shown acceptable reliability and validity for eliciting consumer perspectives on the overall quality of care (Howard et al., 2003). The MHSIP provides a rating scale on an acute hospital environment, exploring the satisfaction of their experience while being treated. This may include their interactions with staff. This was important to understand consumer satisfaction on MHIUs and whether this meets what service is currently being delivered. A total of 14 items from the MHSIP were included in the online survey as they were deemed the most relevant to the research question. Seven items were excluded from the original MHSIP, including questions such as, “staff returned my calls within 24 hours,” which is not relevant to inpatient care. The internal consistency of the MHSIP was calculated in the study by Howard et al. (2003) using Cronbach’s alpha (.96).

The second tool was the CLIP (Levick, Broome, Oprescu, et al., 2023). The CLIP was designed to explore current, past, and desirable activities with consumers to assist with goal setting and therapeutic intervention. This checklist was adapted from the Modified Interest Checklist (MIC) (Kielhofner & Neville, 1983) and explored the interests of consumers within the past year. The CLIP (Levick, Broome, Oprescu, et al., 2023) was developed by the authors to elicit information about leisure interests and participation across a comprehensive range of contemporary activities. In developing this checklist, previous studies identified good reliability (*n* = 295 healthy controls, Cronbach’s alpha = .853) and good validity (*n* = 14 practicing occupational therapists).

The LBS is also considered to be a valid and reliable tool (Iso-Ahola & Weissinger, 1990). The LBS is designed to understand consumer satisfaction with the level of engagement and opportunity of activities available in their environment. Iso-Ahola and Weissinger (1990) conducted three studies to reach this conclusion of good reliability (Cronbach’s alpha = .85, .88, and .86).

### Procedures

All recruitment was in the MHIUs at the Princess Alexandra Hospital. Consumers in these locations were acutely unwell with severe and complex mental health issues. All of these locations were considered “locked” and there was a mixture of voluntary and involuntary consumers (Queensland Government, 2016).

Initially, posters were placed in all the MHIUs with a QR code asking for volunteers to complete the survey. There was little uptake with this method, so consumers were directly offered the opportunity to participate with an electronic tablet. Many consumers asked for a reward for participating and opted to not engage when learning there wasn’t one. Consumers’ capacity to participate was assessed by nursing staff on the MHIUs in conjunction with the first author. Consumers completed the survey at their own pace through an electronic tablet or on their own device.

### Data Analysis

Statistical analysis of participant demographic information and questionnaires was analyzed through Statistical Package for the Social Sciences (SPSS) version 26. Internal consistency was completed for the MHSIP and CLIP using Cronbach’s alpha. Statistical significance was determined by paired *t*-tests.

Qualitative data which included the open-question responses were analyzed through content analysis in Microsoft Excel. Content analysis was chosen to identify like concepts and themes in the data (Graneheim & Lundman, 2004). Raw data was placed in a meaning unit category and further condensed or paraphrased. The first author then coded the condensed meaning units into categories and then like themes.

Primary descriptive statistics assisted to analyze like terms or frequency of concepts such as suggested activities by participants. These responses were tabulated and concept counting occurred.

All variables were tested to determine potential associations. Associations were conducted against like variables, for example, satisfied versus dissatisfied and engaged versus disengaged. All questions that explored these factors were analyzed using Somers’d in SPSS (Newson, 2002). All surveyed questions from all tools were analyzed individually for potential associations.

Rigor was enhanced through the use of an audit trail and “critical friend” (i.e., review of coding by other authors) methods during the content analysis of qualitative data (Deuchar, 2008; Graneheim & Lundman, 2004). All analyses were conducted by J.L. and independently analyzed by K.B. Remaining authors reviewed the analyzed data and provided a critical lens to the data set. All feedback was considered by the group and included if a majority deemed important or relevant. All survey responses were anonymous to enable participants the opportunity to freely describe their experiences, perspectives, and opinion. The researcher did not have a relationship with consumers, which may affect the credibility of the findings.

## Results

### Participants

The survey was completed with 57 partial responses by participants and 41 completed responses. Partial responses included consumers who entered the survey but had spent less than 48 hr in the inpatient unit, so the survey ended after Question 2. Other partial responses were due to consumers entering the survey and stopping. On average, the survey took 14 min and 30 s to complete. All participants identified they were in Brisbane, Queensland, Australia. Participants identified as female (51.52%), male (45.45%), and other (3.03%). Most consumers were between the age of 18 and 24 (34.38%), followed by 25–34 (21.88%), 35–44 (25%), 45–54 (9.38%), 55–64 (6.25%), and 65+ (1%).

Participants indicated their understanding of their diagnosis through multiple choices. Responses included depression (18.75%), anxiety such as generalized anxiety and obsessive-compulsive disorder (15.63%), personality disorders such as borderline type (6.25%), schizophrenia (6.25%), schizoaffective (3.13%), bipolar affective disorder (25%), other mood disorders (6.25%), other psychotic disorders (9.38%), and none of the above (9.38%). Participants could also include a free-text option. Some of the written responses included “human” (1), “opinionated” (1), “anorexia nervosa” or “eating disorder” (4), “post-partum depression” (1), “paranoia” (1), “mania” (1), and “ADHD” (1).

### Satisfaction

A majority of participants reported to be either “very satisfied” (24.24%) or “somewhat satisfied” (36.36%) with the level of activity offered when asked in a multiple-choice question beginning of the survey. The remainder of consumers were “neither satisfied nor dissatisfied” (15.15%), “somewhat dissatisfied” (12.12%), or “very dissatisfied” (12.12%).

There was a statistical significance between participants who selected disagreed with the statement “if I had choices, I would still get services from this agency” and “I am better able to do the things I want to do” in the MHSIP (*t* = 3.426, *p* < .001). This was also relevant for the association between participants who disagreed with “I liked the services I received there” and “I am better able to do the things I want” (*t* = 3.577, *p* = .001) in the MHSIP. Similarly, participants who reported being dissatisfied with the level of activity offered (in the MHSIP) also reported being unable to engage with the activity available (in the LBS) (*t* = 3.677, *p* < .001).

### Consumer Perspectives on Occupational Opportunities

A majority of consumers reported leisure to be of high value to them. Participants rated the value based on a sliding scale from 0 to 100 (*M* = 79, *SD* = 27). In free-text options indicated they were “bored” or “there’s nothing to do.” Participants identified some of the current activities available included “walking the hallways,” “talking to others,” “basketball,” “listening to music,” “watching television,” “board games,” and utilizing their mobile phones for activities such as Netflix (television streaming service) or games. On average, participants identified three activities currently offered on the MHIUs.

A summary of the results for the MHSIP is collated in Table 1 and the LBS in Table 2. Responses have been categorized as agree (“strongly agree” or “agree”), neutral, or disagree (“strongly disagree” or “disagree”). The MHSIP achieved high internal consistency (*M* = 53.5, *SD* = 12.52, Cronbach’s alpha = .805). A summary of the CLIP can be seen in Table 3. Internal consistency was also considered high in the CLIP (*M* = 184.59, *SD* = 52.419, Cronbach’s alpha = .96).

**Table 1.** Mental Health Statistics Improvement Program Results.

MHSIP Question	Agree (%)	Neutral (%)	Disagree (%)
Satisfaction			
If I had other choices, I would still get services from this agency.	10 (31.3)	14 (43.8)	8 (25)
I liked the services that I received there.	10 (31.3)	16 (50)	6 (18.8)
I would recommend this agency to a friend or family member.	8 (25)	14 (43.8)	10 (31.3)
Access			
Staff were willing to see me as often as I felt it was necessary.	10 (31.3)	17 (53.1)	5 (15.6)
Services were available at times that were good for me.	8 (25)	19 (59.4)	5 (15.6)
I was able to get all the services I thought I needed.	6 (18.8)	18 (56.3)	8 (25)
Appropriateness			
I was encouraged to use consumer-run programs (support groups, drop-in centers, crisis phone line, etc.).	7 (21.9)	16 (50)	9 (28.1)
Functioning			
I did things that were more meaningful to me.	11 (34.4)	15 (46.9)	6 (18.8)
I am better able to take care of my needs.	8 (25)	19 (59.4)	5 (15.6)
Outcomes			
I deal more effectively with daily problems.	8 (25)	18 (56.3)	6 (18.8)
I deal better in social situations.	9 (28.1)	21 (65.6)	2 (6.3)
My symptoms are not bothering me as much.	10 (31.3)	17 (53.1)	5 (15.6)
I am better able to do things that I want to do.	9 (28.1)	17 (53.1)	6 (18.8)
Participation			
I, not staff, decided my treatment goals.	8 (25)	15 (46.9)	9 (28.1)

**Table 2.** Leisure Boredom Scale Results.

Criteria	Agree (%)	Neutral (%)	Disagree (%)
For me, leisure time just drags on and on.	8 (25)	10 (31.3)	14 (43.8)
During my leisure time, I become highly involved in what I do.	21 (65.6)	9 (28.1)	2 (6.3)
Leisure time is boring.	4 (12.5)	10 (31.3)	18 (56.3)
If I could retire now with a comfortable income, I would have plenty of things to do for the rest of my life.	19 (59.4)	8 (25)	5 (15.6)
During my leisure time, I feel like I’m just “spinning my wheels”	8 (25)	14 (43.8)	10 (31.3)
In my leisure, I usually don’t like what I’m doing, but I don’t know what else to do.	8 (25)	12 (37.5)	12 (37.5)
Leisure time gets me aroused and going.	15 (46.9)	14 (43.8)	3 (9.4)
Leisure experiences are an important part of my quality of life.	23 (71.9)	7 (21.9)	2 (6.3)
I am excited about leisure time.	18 (56.3)	12 (37.5)	2 (6.3)
In my leisure time, I want to do something, but I don’t know what to do.	14 (43.8)	12 (37.5)	6 (18.8)
I waste too much of my leisure time sleeping.	9 (28.1)	9 (28.1)	14 (43.8)
I like to try new leisure activities that I have never tried before.	18 (56.3)	10 (31.3)	4 (12.5)
I am very active during my leisure time.	16 (50)	9 (28.1)	7 (21.9)
Leisure time activities do not excite me.	5 (15.6)	10 (31.3)	17 (53.1)
I do not have many leisure skills.	12 (37.5)	7 (21.9)	13 (40.6)
During my leisure time, I almost always have something to do.	18 (56.3)	11 (34.4)	3 (9.4)

**Table 3.** Results From Checklist of Leisure Interests and Participation Based on Consumer Interests in the Past Year.

Activity	I currently do this, %	I don’t do it anymore, but I’d like to, %	I have never done it, but I’d like to, %	I don’t do this, and I don't want to, %	Personally, I don’t consider this leisure, %
Adventure activities (e.g., climbing, gliding, surfing, skateboarding)	22.58	25.81	19.35	19.35	12.90
Animal Husbandry (e.g., beekeeping)	9.68	19.3	29.03	29.03	12.90
Art/Craft	38.71	22.58	12.90	19.35	6.45
Athletics (e.g., running, track and field)	29.03	22.58	6.45	25.81	16.13
Babysitting	19.35	12.90	9.68	29.03	29.03
Board/card games	54.84	12.90	9.68	12.90	9.68
Camping	22.58	38.71	16.13	9.68	12.90
Checkers/Chess	35.48	19.35	9.68	22.58	12.90
Circus/aerial acrobatics	10.00	13.33	23.33	36.67	16.67
Coloring-in	53.33	10.00	10.00	13.33	13.33
Computer-related activities (e.g., games, internet browsing)	70.00	10.00	10.00	10.00	0.00
Concerts/festivals	33.33	43.33	10.00	10.00	3.33
Cooking/baking	56.67	23.33	6.67	10.00	3.33
Cosplay	10.00	0.00	20.00	40.00	30.00
Cultural activities	33.33	16.67	23.33	16.67	10.00
Cycling	20.00	20.00	16.67	20.00	23.33
Dancing	30.00	26.67	16.67	16.67	10.00
Dating	20.00	10.00	26.67	20.00	23.33
Do it yourself activity	56.67	3.33	20.00	10.00	10.00
Driving	51.72	17.24	13.79	0.00	17.24
Eating out with friends	41.38	20.69	13.79	13.79	10.34
Exercise/fitness/gym	48.28	17.24	17.24	10.34	6.90
Fishing	10.34	10.34	20.69	13.79	44.83
Foreign languages	31.03	6.90	13.79	17.24	31.03
Gardening yard work	31.03	24.14	20.69	10.34	13.79
Going for a walk or run	75.86	10.34	6.90	3.45	3.45
Going to a party	48.28	20.69	10.34	10.34	10.34
Hairstyling/Makeup	27.59	13.79	24.14	17.24	17.24
Hiking	24.14	27.59	13.79	10.34	24.14
Homebrewing	6.90	6.90	20.69	24.14	41.38
Home decorating	20.69	48.28	17.24	6.90	6.90
Horse riding	10.34	34.48	34.48	6.90	13.79
Ice skating	13.79	27.59	24.14	17.24	17.24
Individual sports (e.g., golf, tennis)	25.00	35.71	17.86	7.14	14.29
Knitting/sewing/crocheting	13.79	20.69	13.79	20.69	31.03
Listening to music	72.41	10.34	13.79	3.45	0.00
Martial arts	13.79	13.79	27.59	20.69	24.14
Meditation	31.03	20.69	17.24	17.24	13.79
Motorsports	10.34	17.24	13.79	20.69	37.93
Movies	65.52	17.24	6.90	6.90	3.45
Painting/drawing	44.83	20.69	6.90	13.79	13.79
Pets/livestock	34.48	24.14	13.79	6.90	20.69
Photography	41.38	20.69	13.79	10.34	13.79
Puzzles	48.28	3.45	13.79	20.69	13.79
Religious activities	34.48	6.90	10.34	20.69	27.59
Renovating	24.14	24.14	17.24	13.79	20.69
Running/jogging	34.48	17.24	10.34	10.34	27.59
Sailing	13.79	6.90	24.14	20.69	34.48
Scrapbooking/card making	20.69	20.69	13.79	17.24	27.59
Sexual activities	44.83	20.69	6.90	6.90	20.69
Shopping	57.14	17.86	17.86	0.00	7.14
Singing	41.38	13.79	17.24	10.34	17.24
Social clubs	37.93	10.34	27.59	13.79%	10.34
Social networking (e.g., Facebook, Twitter, Instagram)	62.07	6.90	6.90	10.34	13.79
Social visit with a friend	72.41	13.79	6.90	6.90	0.00
Table tennis/pool	44.83	24.14	17.24	6.90	6.90
Tai Chi	20.69	6.90	17.24	27.59	27.59
Team sports (e.g., soccer, basketball, hockey, football)	27.59	27.59	10.34	17.24	17.24
Television	58.62	10.34	6.90	13.79	10.34
Vacation	53.57	25.00	10.71	0.00	10.71
Vehicle restoration	17.24	10.34	13.79	20.69	37.93
Video games (e.g., PlayStation, Xbox)	37.93	6.90	6.90	20.69	27.59
Visiting a museum	31.03	17.24	10.34	17.24	24.14
Volunteer services	24.14	31.03	17.24	6.90	20.69
Water activities (stand-up paddleboarding, kayaking)	17.24	17.24	27.59	17.24	20.69
Water sports (e.g., swimming, water polo, diving)	31.03	20.69	10.34	10.34	27.59
Woodwork/mending/fixing	17.24	17.24	20.69	10.34	34.48
Writing	34.48	17.24	13.79	20.69	13.79
Yoga/pilates	24.14	24.14	17.24	17.24	17.24

### Barriers

Participants were provided a sliding scale (rated from 0 indicating “limited activity” to 100 indicating “a lot of activity”) on their ability to currently engage in leisure activity on MHIUs (*M* = 45, *SD* = 31). Some of the barriers suggested by participants that prevented them from engaging in leisure activity included lack of motivation, drowsiness or sedation, no one to do an activity with, poor attention span, staff limitations or restrictions (i.e., not enough staff, or eating disorder consumers not being allowed to engage in activity), and time.

A majority of participants indicated there was limited meaningful activity to do on the weekend or outside of business hours. Four participants reported there was limited activity to engage in with eating disorder–related issues. On average, participants provided one to two barriers they could identify that prevented them from engaging in activity. Participants typically indicated the factors preventing them from engagement were either internal (e.g., motivation, mental illness, sedation) or external factors (e.g., environment, time, lack of activity offered, mental health act). An equal number of participants indicated internal and external factors as barriers to engagement which aligns with the findings from Bowser et al. (2018).

### Facilitators

Participants provided feedback, in the free-text options of the survey, for leisure activities they would like to see in the MHIUs. Suggestions included group sessions to improve coping strategies; cooking groups (which would assist to improve community-based skills); gardening groups (this could have a sensory informed approach with herbs and flowers); music in the courtyard; increasing the number of group sessions per day (to more than one); independent activity resources (such as pencils, coloring-in books, sudoku, crosswords, word searches, chalk); “game nights” such as bingo or trivia; photography; and golf. This is consistent with the findings from the CLIP (see Table 3). 53.33% of participants indicated they currently engage in coloring in, 10% stated they “don’t this but they’d like to,” and 10% said “I have never done this, but I’d like to.” Seventy percent of participants indicated they enjoy computer games. Ninety-three percent of consumers indicated they enjoy social visits with friends, and they currently or would like to do this. The remainder of the feedback (46% of participants) provided one to two suggestions such as more activity or more engagement with staff.

The majority of suggestions were either independent activities (such as coloring in or gardening) that could also be potentially undertaken in parallel with others, or social activities that provided structured social interactions with clear rules and turn-taking (e.g., group sessions, golf, bingo). Potentially, this may reflect the capacities of participants to independently take part in more unstructured social activities.

## Discussion

The first aim of this research was to understand the current context of leisure opportunities available to consumers in Australian MHIUs. This study supported findings from Bowser et al. (2018) that there are multiple factors that affect consumer engagement in leisure activity on MHIUs. Several barriers were listed by participants including staff (time availability and shortages), limited range of activities beyond crafts, and lack of activity beyond business hours, to name a few. This was particularly highlighted in the findings of the MHSIP (Table 1) and LBS (Table 2) tools. Participants tended to agree (34.4%) or provided a neutral (46.9%) response to question “I did things that were more meaningful to me” on the MHSIP. This may be due to ambivalence around what is offered or may indicate that internal factors are a larger issue than the limited activities offered.

It was hypothesized that consumers would report dissatisfaction while admitted and a high level of boredom. Participants who indicated they were dissatisfied with the level of activity (MHSIP) also reported they were unable to engage in activity available (LBS) (*t* = 3.677, *p* < .001), which rejects the null hypothesis. There was some disparity between what was reported in the free-text boxes and what participants indicated on the tools or checklists (such as the MHSIP). Some of the consumers reported they enjoyed the lack of stimuli or need to engage in activity as this supported their ability to improve in their mental state. Others suggested that this was a barrier for their recovery. Acute mental illness can also include cognitive comorbid difficulties, mood disturbance, and psychosis, which can affect a person’s ability to engage in meaningful occupation (Marshall et al., 2020). Meaning and purpose are key considerations to participate and engage in day-to-day occupations to provide fulfillment and enrich participation (Law, 2002). Over the past 20 years, despite growing evidence linking restrictive environments, boredom, and poorer mental health (Bowser et al., 2018; Cutler et al., 2021; Howard et al., 2003), there appear to be little change in occupational opportunities provided in locked (mental health or forensic) settings.

Shaffer et al. (2022) indicated that consumer satisfaction reported through the MHSIP was typically correlated to social connectedness and functioning. This was supported by the findings in this study, where participants identified there was a lack of social engagement from their co-consumers and staff. As participants were acutely unwell, it may be of benefit for face-to-face interviews, focus groups, or post discharge exploration of their reflection from their experience. The results indicated that a majority of participants were moderately satisfied with the leisure activity on offer, but internal factors (motivation, boredom, and sedation) prevented engagement, while largely external factors such as a lack of support, and time (from staff to provide activity) prevented engagement. The findings of this study are consistent with Howard et al. (2003), which suggest that consumers are typically less satisfied and bored than the general adult population. There has been limited research utilizing the MHSIP or the LBS in Australian MHIUs to generalize the data found in this study. Marshall et al. (2020) confirm the lack of quantitative approaches to participation and boredom in MHIU populations. Furthermore, this review highlights the lack of empirical evidence associated with the association of boredom and prolonged hospitalization in mental health settings.

When participants completed the MHSIP, most consumers reported they were dissatisfied with the leisure activity available on the MHIU (average rating of 4.5 out of 10 on leisure availability with 0 indicating no opportunity). This was a contrasting response to an initial question on the survey, where participants stated they were satisfied with the leisure activity offered. Some of the reasons for a reduced score may be better understanding of the second question, or cognitive fatigue of answering questions in a survey. Participants may have observed activity occurring on the MHIUs but did not classify this as leisure which may have contributed to contrasting responses.

The second research question aimed to explore the barriers and facilitators to leisure engagement in MHIUs. Each tool was selected to explore potential barriers (MHSIP and LBS) and facilitators (CLIP) to participation on MHIUs. Participants highlighted difficulty engaging in activities with limited people able or willing to enjoy activities. Participants also indicated there were multiple internal factors that are barriers to engagement boredom. This was also demonstrated during the recruitment of surveys. Participants were more likely to complete the survey if someone was assisting them and facilitating the activity. Participants listed some barriers to participating, including drowsiness and lack of motivation. An important finding during the data collection and reports from participants was consumers were more likely to engage when encouraged or assisted. Therefore, regardless of the activities on offer, consumers may be more likely to engage in activity with prompting or someone to participate with.

The final research question aimed to understand the occupational opportunities available to consumers in Australian MHIUs. The findings of this study were consistent with current body of literature suggesting that consumers are bored and would benefit from better occupational opportunities (Bowser et al., 2018; Folke et al., 2018; Marshall et al., 2020). Consumers report a need for greater support from staff and improved social connectedness. Consumers also reported a barrier to engagement was the built environment and more time allocated from staff. The findings by Wilson et al. (2018) suggested the need to review the role of staff, the built environment, and the need to provide occupational opportunity on MHIUs. Often nursing staff report they are inundated with their documentation and other responsibilities, which reduces their capacity to engage with consumers in a meaningful capacity (Whittington & McLaughlin, 2000). If the external environment is more conducive to recovery, boredom and internal factors inhibiting participation can be adequately assessed and targeted (Marshall et al., 2020).

Consumers should be provided with occupational opportunity that facilitates recovery and engages them. The CLIP was used to gain tangible leisure preferences from consumers to understand what leisure activity could be offered on MHIUs. The CLIP provided insight into leisure interests that most of the participants reported being interested in (Table 3). A recommendation for some activities that could be offered has been included to provide services an opportunity to explore what they currently offer and potential resources.

### Limitations

This study had some limitations but overall achieved the aims of the study. The data in this study were collected at one hospital in Brisbane, Queensland, Australia, therefore the results may not be generalized to all MHIUs or the consumer population in Australia. Furthermore, convenience sampling may have led to potential bias. Consumers were considered acutely unwell while completing the surveys which may have influenced their perspective of the services, and it may differ post hospitalization. A limitation of this study was consumers’ length of hospitalization (days), diagnosis, treatment modalities, and mental health act status were not collected, so consumers with lengthy hospitalizations may have reported less variety of activity than other consumers. Potential confounding factors (such as education level, acuity, previous occupational history, socioeconomic status, and typical environment) may have contributed to selection bias of activities on the CLIP and the suggested activities in the qualitative data. The survey was lengthy which may have contributed to many consumers not completing the entire survey.

### Future Research

Participant uptake was low unless consumers were directly asked and offered a device to complete the survey, some of which required support to use the device due to acuity and skill level. Future research may review the data collection method for a higher uptake of responses and consider face-to-face interviews. Participants requested a reward for participation which may assist with recruitment. Remuneration of $AUD may be considered in future ethics applications as a small payment for engagement as there is no direct benefit to engagement otherwise. A deeper exploration of leisure activities that consumers with an eating disorder can participate in would provide more occupational opportunities for this cohort.

## Conclusion

Leisure activity is an often undervalued therapeutic modality within mental health. During consumers’ admission, engagement in occupation in an inpatient environment can reduce the need for acute medication use, minimize aggressive incidents that require seclusion (Kontio et al., 2012), and increase the therapeutic alliance with staff.

Harnessing a person’s interest in leisure activity can be health creating, a concept aligned with the health promotion principle of salutogenesis (building peoples’ capacities and resources to improve health) (Caldwell, 2005; Lee & Hwang, 2018). The use of standardized tools and checklists can help therapists to build an occupational profile as well as identify opportunities for an enhanced leisure profile to support therapeutic goals.

## Key Points for the Multidisciplinary Team

- Consumers have the capacity to report their interests to engage in meaningful activity that affects their care.
- Standardized tools and checklists are a suitable and helpful way to assess the leisure interests of consumers on acute MHIUs.
